# Pregnant women have inadequate fiber intake while consuming fiber‐rich diets in low‐income rural setting: Evidences from Analysis of common “ready‐to‐eat” stable foods

**DOI:** 10.1002/fsn3.1188

**Published:** 2019-09-05

**Authors:** Taddese Alemu Zerfu, Aleme Mekuria

**Affiliations:** ^1^ College of Health Sciences Dilla University Dilla Ethiopia; ^2^ Maternal and Child WellBeing (MCW) Unit African Population and Health Research Center Nairobi Kenya; ^3^ Department of Public Health Arbaminch College of Health Sciences Arba Minch Ethiopia

**Keywords:** adequacy, fiber, pregnancy, pregnant women, trimester

## Abstract

The intake of fiber‐rich foods during pregnancy has several health benefits to the pregnant woman including lowering the risks of diabetes, preeclampsia, and constipation. However, little is known about the content, daily intake levels, and adequacy of fiber among pregnant women in many low‐income settings. We aimed to identify common food items and determine adequacy of dietary fiber intake levels among pregnant women in rural Ethiopia. Dietary data were collected from a subsample (*n* = 55) of pregnant women enrolled to a larger prospective cohort study (*n* = 414). Dietary intake level was measured using repeated 24‐hr dietary recall method and weighing the total amount of daily food. Fiber content was determined using the Weende method supplemented by other sources.The mean [±*SD*] dietary fiber intake level was 25.89 [±5.09 mg/g] per day, which declined across pregnancy trimester from 26.01 [±9.18 mg/g] to 22.67 [±9.01 mg/g] and 24.56 [±9.98 mg/g] during the first, to second and third pregnancy trimesters, respectively. Boiled cereals and coffee contributed to nearly two‐thirds (63.2%) of the daily fiber intake, while the major bulk of daily food, enjera, contributed to less than a quarter (24.3%) of the fiber intake. Though dietary patterns favor diversified intake for fiber, the mean intake levels below the recommended levels and proportion of women getting adequate to the physiologic stages of pregnancy were inadequate compared to the standard. Mothers, in such community, could benefit from increasing overall intake of existing food to satisfy their fiber needs during pregnancy.

## 1 INTRODUCTION

Maternal diet during the preconception, pregnancy, and lactation has been linked to the immediate and long‐term prenatal outcomes (Allen, 2000; Christensen, Leach, & Mackinnon, 2010; Grieger, Grzeskowiak, & Clifton, 2014; Muslimatun, 2001; Noronha, Al, Seshan, Ramasubramaniam, & Raman, 2012). During this period, the essential building blocks for brain development, healthy growth, and a strong immune system are founded for continued development throughout life (Pem, 2017). A growing body of scientific evidence also shows that some of the foundations of a person's lifelong health risks and predisposition to chronic diseases are largely set during this period (Mameli et al., 2016). During the periods of pregnancy and lactation, the need for macro‐ and micronutrients as well as dietary fiber increases sharply to support health pregnancy and childbirth (Potdar et al., 2014; Stephens, Payne, Ball, Pencharz, & Elango, 2015).

Increasing intake of dietary fiber has exceptionally been linked to several additional benefits even compared to nonpregnancy state and the general adult population (Anderson et al., 2009). This is mainly attributable to physiologic changes taking place and the raise in the level progesterone hormone that causes heartburn, constipation, and indigestion during pregnancy (Anderson et al., 2009; Williamson, 2006).

Dietary fiber is a collective term for a variety of plant substances that are resistant to digestion by human gastrointestinal enzymes with complete or partial fermentation in the large intestine. It includes polysaccharides, oligosaccharides, lignin, and associated plant substances. Fiber is found only in plant foods like fruits, vegetables, legumes, and whole grains. Animal products such as meat, milk, and eggs do not contain fiber (Summary E, Definition DF 2001).

The intake of fiber‐rich foods during pregnancy and lactation helps in promoting heart health, decreasing diabetes risk, preventing constipation, as well as reducing risks of preeclampsia risks, together with providing nutrient‐rich food with low energy density (Anderson et al., 2009; Camey et al., 2008). Dietary fibers also promote beneficial physiological effects lowering the risk of coronary heart disease, stroke, hypertension, diabetes, obesity, and certain gastrointestinal diseases. Increasing fiber intake also lowers blood pressure and serum cholesterol levels.(Anderson et al., 2009; Camey et al., 2008; Theuwissen & Mensink, 2008) and even reduced risk of mortality (Kim and Je, 2014). Among pregnant women with type 1 diabetes, higher fiber intake is associated with lower daily insulin requirements (Kalkwarf, Bell, Khoury, Gouge, & Miodovnik, 2001).

Despite clear health benefits, little is known about intake levels and food items contributing to intakes. In resource‐poor settings like rural Ethiopia, diets mainly originate from plant sources, which are known to be rich sources of dietary fiber. Foods were prepared from cereals, grains, and legumes making majority of the daily intakes (Amare et al., 2012; Ethiopian Health & Nutrition Research Institute, 2009; Mesfin Welde mariam, 1985). However, to the best of our knowledge, no ever study has investigated the daily intake levels and adequacy of dietary fiber, particularly among the most vulnerable population groups, like pregnant mothers. This study aims to identify fiber content of common food items and quantify intake levels and adequacy among pregnant women in rural Ethiopia.

## 2 METHODS

### 2.1 Study setting

The study was conducted in rural central Ethiopia, Arsi Zone, Oromia region. This area is among the leading producers of wheat and barley in Ethiopia. The Zone is divided into four agro‐climatic areas mainly due to variation in altitude. Arsi Zone is among the surplus crop‐producing areas in Ethiopia (Oromia Bureau of Finance and Economic Development (OBOFED) 2015). According to the latest (2015) national population projection based on the population and housing census, the Zone had over 2, 635,515 population. It is dominated by rural (89%) population, whereby females accounting for 52.85% of the urban and 49.94% of the rural population.

Arsi Zone has great diversity in altitude and physiographic diversity. Accordingly, there are four major identified physiographic divisions: cool, temperate, warm, and lowland (“Kola”). The cool agro‐climatic zone ranges an altitude of above 3,500 m above sea level (masl) covering the highest altitudes areas of the zone, but has only a share of 2.74% of the total area in the zone. The cool temperate agro‐climatic zone that includes the mountain ranges massifs and high plateaus ranging between 2,500 and 3,500 masl and lies in the central part of the zone, stretching from the border of the southern region of Ethiopia to Aseko District and belongs to the Arsi‐Bale Massifs. This makes about 22.74% of the total area of the zone. The warm temperate agro‐climatic zone that ranges between 1,500 and 2,500 masl comprises low plateaus of the zone and covers about the 49.60% of zonal while lowlands constituting a quarter (24.92%) of the total area of the zone. The physiographic region of the zone is similar to the Awash River valleys and southeastern lowlands. In general, the zone has the lowest altitude in extreme east of Seru District located in Wabe gorge which is 805 masl and highest point on peak of mount Kaka which is 4,195 masl (Oromia Bureau of Finance and Economic Development (OBOFED) 2015).

Arsi Zone produces a number of different varieties of agricultural crops ranging from cereals to pulses, vegetables, fruit, oilseeds, and spices. Crop production by area is predominantly cereals followed by pulses, vegetables, oilseeds, and fruit crops. The Zone is referred to as surplus grain‐producing areas in the country.

### 2.2 Study subjects

For the determination of socio‐demographic characteristics, nutritional data, dietary patterns, and identification of common food items consumed in the study area, we included randomly selected pregnant women (*n* = 418) visiting antenatal care services from eight rural health centers of Arsi Zone between August 2014 and March 2015. Being part of a larger study focusing on anemia risks during pregnancy (Nyambose, Koski, & Tucker, 2002), the sample size was calculated by use of the Open Epi Kelsey statistical software available at with the following parameters and assumptions: a 95% significance level (2‐sided), 80% power, and 37% anemia prevalence among exposed pregnant women and an anticipated 10% lower prevalence of anemia among unexposed pregnant women. This yielded a total of 168 subjects/arm, and to allow for ≤20% attrition by the end of the study, a sample size of 420 was required. Pregnant women who are permanent residents of the study area, with no known medical, surgical, or obstetric problems, and who were willing to attend routine ANC visits were included into the study. All relevant dietary, nutritional, anthropometric, and health data were collected every month for at least four visits.

### 2.3 Dietary Assessment

Dietary intake data were collected using repeated 24‐hr dietary recall of pregnant mothers and a 3‐day weighed food record [weighing the total amount of daily food intake] in a subsample (*n* = 55) of the study population. The weighed food record is often regarded as the most precise method for estimating the food and nutrient intakes of individuals. This approach adopts the same methodological principles as the estimated food record method. The method has been shown to be useful in collecting information for different purposes like collecting dietary data on group mean intakes, where a single record is sufficient, or to measure the distribution of individual intakes, where multiple record days are needed. The method has also been validated for use on pregnant subsistence farmers in various parts of a low‐income setting (Nyambose et al., 2002).

For the 24‐hr dietary recall, an interactive quantitative recall was conducted using the technique adapted and validated for use in developing countries (Gibson & Ferguson, 1999). The respondents were asked to recall the exact food intake of the previous day. They were also asked to list the detailed descriptions of all foods including recipes and beverages consumed and to estimate portions and quantities based on pictures and locally used measuring tools like cups or teaspoons.

All days of the week were equally represented in the sample. Midwives assisted by other experienced data collectors were locally recruited and trained in a classroom setting. This was followed by a pilot test on a group comparable to that of the actual study.

Data were collected considering the two distinct seasons (preharvest of the main harvest season, which occurs commonly between August and October and the peak harvest season, November–January) in the area, which can have impact on the results and interpretations.

### 2.4 Analysis of total dietary fiber intake/consumption

**Figure 1:**
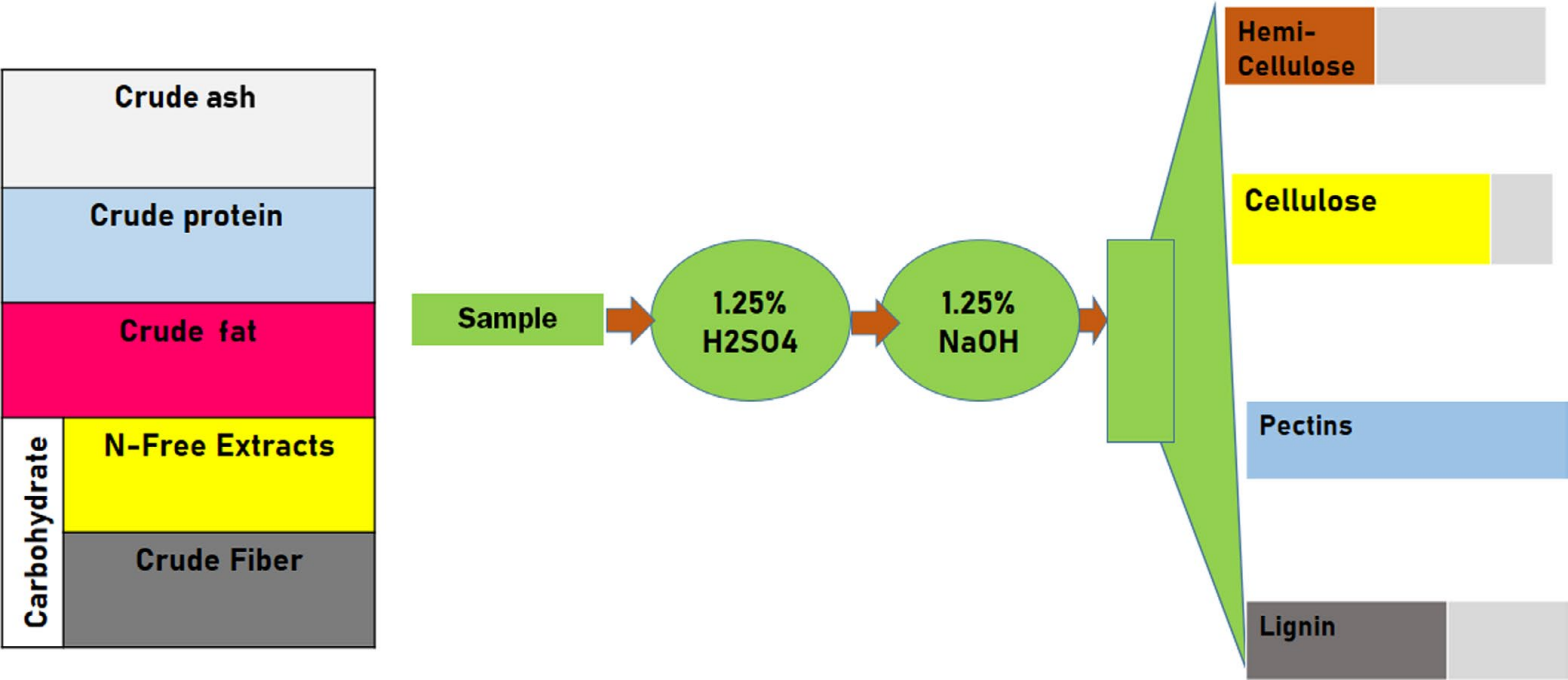
Crude fiber analysis according to the Weende method

Determination of the level of dietary fiber was conducted following the Weende method modified by Hum at the laboratories of Ethiopian Public Health Institute (EPHI), Food and Nutrition department. Some missing values were compiled from the Ethiopian food composition tables’ database, after adjustment of moisture content. The laboratory analysis was commenced by weighing 2 g of samples from each food item in a 600‐ml beaker and adding 200 ml 1.25% H2SO4a and boiling it for 30 min placing a watch glass over the mouth of the beaker. It was then heated gently on hot plate keeping the level constant with distilled water. After exactly 30 min, we added 20 ml 28% KOH and boiled gently for further 30 min, stirring it occasionally. It was then filtered covering the bottom of a sintered glass crucible with 10 mm sand and wetting the layer of sand with a little distilled water, after which the solution poured from beaker into sintered glass crucible and then turned on vacuum pump. All the other filtration and drying and combustion steps were strictly followed. We applied the following formula to calculate the crude finer from the samples (Figure 1).

$$\text{Calculation}\;=\;\text{crude}\;\text{fiber}\;\text{in}\;\text{\textbackslash{}\%}\;=\frac{(W_{1}-W_{2})\times 100}{W_{3}}$$

where *W*_1_—crucible weight after drying in an oven.

*W*_2_—crucible weight after ashing.

*W*3—sample dry weight.

### 2.5 Statistical analysis

The mean ± *SD* daily fiber intake and fiber content of major food items were computed and tabulated. The mean intakes of dietary fiber across various agro‐ecologic areas of the zone were compared by independent‐sample *t* test. Chi‐square test of proportion was used to determine the percentage of participants with intakes at or below the recommended daily allowance and adequate intakes. All statistical analyses were undertaken using SPSS version 13. *p* values <.05 were considered statistically significant.

## 3 RESULTS

Detailed socio‐demographic, anthropometric, and nutritional characteristics of study participants are given in Table 1. Nearly eight in every ten (83.2%) and four in every five (80.5%) of the study participating pregnant mothers were under thirty by age and belong to the Oromo their ethnic group, respectively. Furthermore, a larger proportion (46.3%) of the mothers were malnourished or had a Mid‐Upper Arm Circumference (MUAC) of less than 23 cm.

**Table 1:** Selected socio‐demographic, reproductive, and nutritional characteristics of pregnant mothers in rural Arsi, central Ethiopia

Characteristics	Frequency	Percent
Maternal age, years		
<20	5	9.1
20–24	22	39.3
25–29	19	34.8
≥30	9	16.8
Ethnic group		
Oromo	44	80.5
Amhara	8	13.9
Guraghe	2	4.5
Other	1	1.1
Educational status		
Unable to read and write	18	32.1
Read and write only	14	25.4
Primary education	5	8.3
Secondary or above	19	34.2
Land size		
<1	20	36.4
1–2	10	18.7
>2	15	44.9
Parity		
0	25	44.7
1	16	28.3
2	9	15.8
≥3	6	11.2
ANC visits completed at birth		
One	3	4.6
Two	17	30.3
≥Three	36	65.1
MUAC (cm)		
Malnourished (<23 cm)	25	46.3
Not malnourished (≥23)	30	53.9
Total	55	100

The main food items consumed with the common timing for consumption and fiber contents for each are presented in Table 2. The fiber content of breakfast foods ranged from as low as 0.8 mg/100 g of edible food in porridge to 8.4 mg/100 in hot drinks (coffee and tea). For lunch and dinner, the main dish enjera (made from various grains with legume‐based stews and sometimes from vegetables) had a fiber content of 1.12 mg/100 g of edible portion (Table 3). Snack foods like roasted beans and boiled cereals contributed nearly two‐thirds (63.2%) of the daily fiber intake of pregnant mothers as they contain large (2 g/100 g of edible food portion) fiber.

**Table 2:** Dietary fiber contents of major food items consumed by pregnant mothers, rural Arsi, central Ethiopia

Food items (Local name)	Description of food item	Fiber content (g/100g)	Common timing for Consumption
Enjera			
Teff (Ye fef enjera)	Enjera is a pancake like bread prepared from cereals such as barely (Hordeum vulgare L.), teff (Eragrostis tef), maize (Zea mays L.), wheat (sinde, Triticum vulgare) or a mixture of these cereals which served as a main dish with the stews	1.12	Lunch and Dinner
Wheat (Ye Sindie Injera)			
Barely (ye gebs enjera)			
Mixed (kiyit Injera)			
Bread (Dabo)			
Wheat bread (Dabo)	Unleavened bread prepared from unfermented maize or wheat or mixed	1.3	Breakfast or snack
Barely (Kita)			
Porridge (Genfo)	Porridge is prepared from cereal or mixture of cereals	0.8	Breakfast
Boiled cereals (Ashuk)			
Beans (Yebakela Ashuk)	Prepared by boiling cereals such like wheat, or legumes such as, broad beans (Vicia faba L.), chick peas (shimbira, Cicer arietinum (L.)) or a mixture of the above in water with salt	7.2	Dinner before the main dish as a complement and during coffee ceremony
Bean and wheat (Nifro)			
Stew/ sauce			
Pea (Ater/bakela kik wet)	Stew prepared from ground legumes such as garden or field peas, chickpea, or grass pea Kale, potato by roasting, decortications and grinding the grains, seasoning with spices and then cooking. Served with a main dish	0.9	Lunch and dinner Added to the main dish
Lentil (Misir Wet)			
Bean (Shiro wet)			
Kale (Atikit/Gomen Wet)			
Hot drinks			
Coffee (Buna)	Prepared by boiling roasted coffee beans or tea leaves in a clay pot. Based on availability and considering fasting seasons, milk could be added to it. Salt, if not sugar are confectionaries used for taste	8.4	All the day after the main dish. Tea is mainly with breakfast in some houses
Tea (shay)			

**Table 3:** Typical example of daily food intake and fiber content of the food staffs for pregnant women in rural Arsi, central Ethiopia

Dieting time	Food items	Mean intake per day (gm/100)	Fiber content (mg/100 g)	Total fiber (g/day)
Breakfast	Porridge	1.8	0.8	1.44
	Coffee	0.45	8.2	3.69
Lunch	Enjera	2.9	1.12	3.25
	Shiro (stew)	0.8	0.9	0.72
	Coffee	0.4	8.2	3.28
Dinner	Bean boiled	0.9	7.2	6.48
	Enjera	2.7	1.12	3.04
	Potato stew	0.9	0.9	0.81
	Coffee	0.4	8.2	3.28
Total		11.25		25.97

**Figure 2:**
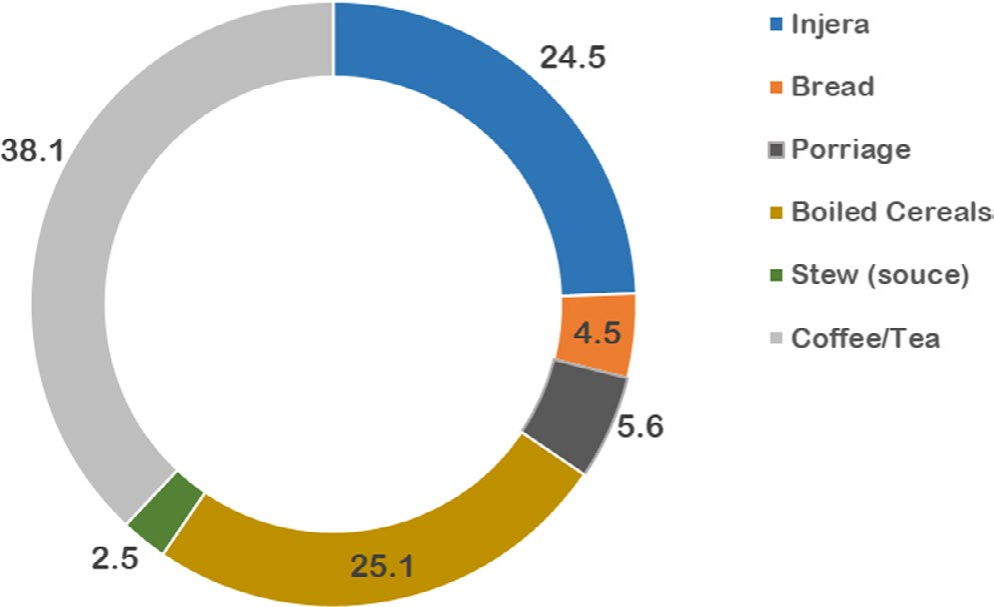
The relative contribution of the various typical food items consumed by pregnant women to the total daily fiber intake

The relative contribution of the various typical food items consumed to the total daily fiber intake is presented in Figure 2. Accordingly, hot drinks like coffee and tea contribute the largest (38%) followed by boiled or roasted cereals that contribute a quarter (25.1%) of the daily fiber intake on average. The major bulk food, enjera, and stew together make nearly one‐third (30.1%) of the daily fiber for mothers during pregnancy.

In Table 4, we presented the average daily dietary fiber requirements and observed intake among pregnant mothers during each trimester of pregnancy. The overall mean ± *SD* dietary fiber intake of pregnant mothers was 25.89 ± 5.09. However, a subgroup analysis across the trimesters of pregnancy trimester showed that the mean intake of dietary fiber significantly declined from 26.01 ± 9.18 mg/g to 22.67 ± 9.01 mg/g and 24.56 ± 9.98 mg/g with progress of in pregnancy trimester from the first to the second and the third, respectively (*p* < .05). This is equivalent to a 5.3 mg/100g (85.7%) and 3.4 mg/100g (66.7%) reduction, compared to the required 28 mg/day.

**Table 4:** Average daily dietary fiber requirements and observed intake of pregnant mothers during each trimester of pregnancy in rural Arsi, central Ethiopia

Pregnancy trimester	RDA (mg/day)	Observed intake mg/per day^a^	Mean deficiency (mg/day)	Percent of mothers below RDA	*p*‐value
First	**28**	26.01 ± 9.18	−1.99	40	<.05
Second	**28**	22.67 ± 9.01	−5.33	85.7	
Third	**28**	24.56	−3.44	66.7	
Total		25.89 ± 5.09	−2.11	60	

Bold: the RDA for dietary fiber remains the same throughout pregnancy.

Abbreviation: RDA, recommended dietary allowance.

a mean ± *SD*

## 4 DISCUSSION

Dietary fiber has several benefits for the pregnant women. It helps the proper digestion during pregnancy, provides important nutrients such as vitamin B groups, helps pregnancy weight gain and to be under control, helps optimal blood glucose control, prevents constipation, prevents cardiovascular disease during pregnancy and also prevents later childhood allergy developments. Accordingly, in this study, we analyzed common “ready‐to‐eat” prepared foods for their fiber content with the aim of measuring adequacy and describing the health implication on pregnancy. We found that the mean ± *SD* daily intake of dietary fibers among the study mothers is 25.89 ± 5.09g/day. This amount of daily intake for fiber is very close to the daily requirement set by the Food and Nutrition Board Dietary Reference Intakes (2011) and also far above the daily intake of urban community in north Ethiopia (Amare et al., 2012). Furthermore, it is far better than the daily fiber intake of an average American and a European women (Du et al., 2010; McGill, Fulgoni, & Devareddy, 2015).

We also found that study subjects (pregnant mothers) mainly relied on food from plant sources originating from cereals and grains as to the main menu of their day‐to‐day diet. Plant food items are known to be excellent sources of dietary fiber, cereals, and grains (Kalkwarf et al., 2001). The Food and Drug Administration (FDA) has also approved that foods high in fruits, vegetables, and whole grain have multiple health benefits for human beings, particularly for pregnant mothers (Lattimer & Haub, 2010). Yet, for majority (60%) of the pregnant mothers studied, the mean intake of fiber is below the minimum amount required which is a controversy to be explained. We attribute two key reasons for why this is happening. First, even if the main foods of the study mothers were from plant sources, the proportion of the mothers with an intake of fruits and vegetables that are ideal sources of fiber is very low. Second, the overall food intake per day per capita is comparably low to provide sufficient amounts of nutrients (EPHI, 2013).

Among the various foods eaten, hot drinks like coffee and boiled beans (locally called “*Ashuk”*) make nearly one‐third of the overall daily fiber intake. The major food that makes the bulk of daily dietary intake, enjera, contributed only a quarter (24.3%) of the daily fiber. It is to be recognized that there are households who have better intake of fruits and vegetables and others with low intake of the hot drinks like coffee and boiled beans in their daily diet. Therefore, this result should be integrated cautiously as it may overestimate the daily fiber intake in some households and/or underestimate in others.

The other critical concern is the deteriorating trend of food intake in general and availability fiber in the diets of pregnant mothers during the critical stages, second and third pregnancy trimester. Physiologically, it is the surge of progesterone levels during pregnancy that affects smooth muscle tone and results in a decreased rate of gastrointestinal transit that ends with constipation. The raise in the level of progesterone during the second and third trimesters of pregnancy worsens and intake for fiber becomes a necessity (Blumfield, Hure, Macdonald‐Wicks, Smith, & Collins, 2012; Brown, 2004; Institute of Medicine, 2002; Kalkwarf et al., 2001). Higher fiber consumption during the second trimester of pregnancy is also associated with reduced insulin resistance for pregnant mothers with family history of type 2 diabetes and/or gestational diabetes.

Our study has limitations. We collected nutritional and other socio‐demographic data from pregnant mothers who visited health facilities in the study area, yet collected food intake from households. Even if we anticipated similar dietary intake among all pregnant women, we know of nothing particularly different in our sample from pregnant women in general seeking care at health centers, and representative studies have not yet been undertaken. Another limitation is we took only 15% of the study population for measuring their daily food intake that is insufficient to get representative and comprehensive food intake data.

In conclusion, we observed that pregnant mothers in the study area had a dietary pattern that favors the adequate intake of foods rich in fiber. Nonetheless, a large majority (60%) of them still have inadequate (<28 g/day) intake of dietary fiber into their daily food. Two food items, boiled cereals and hot drinks like coffee, constituted over two‐thirds of the total daily fiber intake. The very important challenge is the deteriorating trend in the mean intake of fiber when it is demanded in late pregnancies. We also recommended that pregnant mothers have to diversity their food, especially they should improve their daily intake of fruits and vegetables during pregnancy which will further ensure adequate intake of fiber that results in healthier pregnancy and childbirth.

## AUTHORS’ CONTRIBUTIONS

TAZ developed the analysis parameters, developed objectives, and secured support. TAZ also undertook analysis of the. AM assisted in design and statistical analyses and was involved in the write‐up and synthesis of the findings. Both authors read and approved the final manuscript.

## ETHICAL CONSIDERATION

The study was approved by both the Addis Ababa University, College of Natural Sciences and the Oromia Regional Health Bureau Ethics Review Committees. The study conforms to the Declaration of Helsinki. A formal and official cooperative letter was written hierarchically from the region to the zonal health office and then to, district to health centers and finally to kebeles (villages). Prior to undertaking interviews, written consent was obtained from all adults, whereas for enrollment of children permission was obtained from their parents or guardians. The authors declare that they do not have any competing interests.
